# Leaf processing behaviour in *Atta* leafcutter ants: 90% of leaf cutting takes place inside the nest, and ants select pieces that require less cutting

**DOI:** 10.1098/rsos.150111

**Published:** 2016-01-27

**Authors:** Ryan W. Garrett, Katherine A. Carlson, Matthew Scott Goggans, Michael H. Nesson, Christopher A. Shepard, Robert M. S. Schofield

**Affiliations:** 1 Department of Physics, University of Oregon, Eugene, OR 97403, USA; 2 Department of Biochemistry and Biophysics, Oregon State University, Corvallis, OR 97331, USA

**Keywords:** leafcutter ants, *Atta*, leaf processing, foraging, metabolic energy, energetics

## Abstract

Leafcutter ants cut trimmings from plants, carry them to their underground nests and cut them into smaller pieces before inoculating them with a fungus that serves as a primary food source for the colony. Cutting is energetically costly, so the amount of cutting is important in understanding foraging energetics. Estimates of the cutting density, metres of cutting per square metre of leaf, were made from samples of transported leaf cuttings and of fungal substrate from field colonies of *Atta cephalotes* and *Atta colombica*. To investigate cutting inside the nest, we made leaf-processing observations of our laboratory colony, *A. cephalotes*. We did not observe the commonly reported reduction of the leaf fragments into a pulp, which would greatly increase the energy cost of processing. Video clips of processing behaviours, including behaviours that have not previously been described, are linked. An estimated 2.9 (±0.3) km of cutting with mandibles was required to reduce a square metre of leaf to fungal substrate. Only about 12% (±1%) of this cutting took place outside of the nest. The cutting density and energy cost is lower for leaf material with higher ratios of perimeter to area, so we tested for, and found that the laboratory ants had a preference for leaves that were pre-cut into smaller pieces. Estimates suggest that the energy required to transport and cut up the leaf material is comparable to the metabolic energy available from the fungus grown on the leaves, and so conservation of energy is likely to be a particularly strong selective pressure for leafcutter ants.

## 1. Introduction

**Figure 1.**
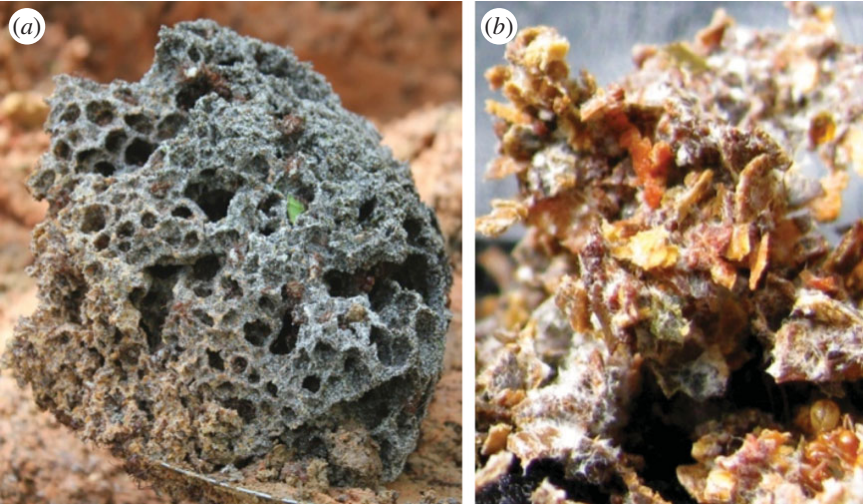
(*a*) Fungal comb excavated from a mature *Atta cephalotes* colony in Minca, Colombia. (*b*) Individual leaf fragments are visible in this sample of fungal substrate from an *Atta colombica* colony, Tiputini, Ecuador.

Ants play a dominant ecological role in nearly every terrestrial environment on Earth [1,2]. Throughout the neotropics, the leafcutter ants are top herbaceous consumers [3,4] and use a unique means of food production that has been described in great detail [3,5–8]. Leafcutter ants of the genus *Atta* are the most derived and prolific of the Attini [9,10]. *Atta cephalotes*and *A. colombica* cut disc-shaped pieces of leaves (leaf discs) from certain plant species, carry them to underground chambers and cut them into many small fragments (we will refer to pieces of leaf discs as fragments). Processed fragments are incorporated into a comb structure (figure 1) and inoculated with the basidiomycete fungus *Leucoagaricus gongylophorus* [11] that serves as a food source as well as a home for brood.

Leaf cutting and carrying are probably the most energetically expensive tasks performed by *Atta* species [12–14]. Although many studies have examined the substrate processing behaviours of *Acromyrmex* and *Atta* species [15–32], none has quantified the cutting that occurs during subterranean leaf processing or the total cutting density (distance cut per square metre of leaf). As energy costs of cutting and carrying are high, and the caloric contents of leaves tend to be low, it is possible that these energy costs are a significant fraction of the caloric profit from the leaves and may set a limit to the distance at which the collection of leaf species with high water content (low calories per mass) is energetically profitable. Here we have estimated values for the ratio of above-ground to below-ground cutting as well as the total cutting density using materials collected in Minca, Colombia (from *A. cephalotes*), and Tiputini, Ecuador (from *A. colombica*), along with leaf-processing observations of our laboratory colony (*A. cephalotes*) at the University of Oregon. In addition, we tested the hypothesis that leafcutter ants prefer to harvest smaller leaves, because smaller leaves require less cutting.

## 2. Methods

### 2.1 Processing observations

We observed and videotaped processing behaviours of our laboratory *A. cephalotes* colony. For ease of recording, we scooped roughly 10 cm diameter balls of comb material and the associated ants into glass-topped containers and provided them with *Rubus armeniacus* or *Prunus lusitanica* leaves. During more than 70 h of leaf-processing observations, videos were made using various consumer cameras mounted on a Zeiss dissecting microscope with custom adaptors. These videos were used to clarify leaf-processing behaviour, and, in particular, to verify that all fragment size reduction was accomplished by cutting with the mandibles (as opposed to chewing with other mouth parts), and to reveal the cutting technique. The videos were also used to set the minimum area required for a fragment to be included in the cutting density calculation (smaller pieces of leaf material in the comb might be from decay of complete fragments).

### 2.2 Estimation of cutting density and ratio

Materials were collected from a mature colony of *A. colombica* near the Tiputini Research Station, Ecuador in May 2009, and from a 2-year-old colony of *A. cephalotes* in Minca, Colombia, in June 2012. The main trunk trails to the selected colonies were identified and incoming plant material was sampled from each trail. We collected every object larger than the ants’ heads that was carried past a line on the trail during several 15-min periods. The colonies were then excavated to retrieve samples of fungal substrate.

**Figure 2.**
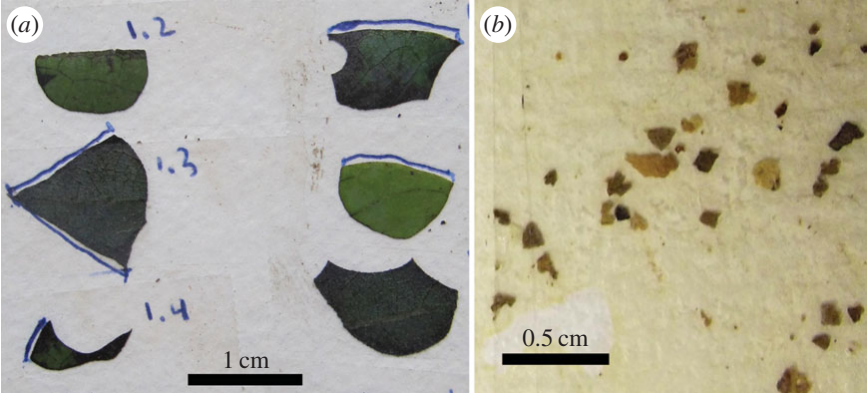
(*a*) Leaf discs collected on foraging trail, Minca, Colombia. The uncut natural edges are marked. (*b*) Leaf fragments collected from fungal comb, Minca, Colombia. Comb material was converted to a slurry by agitating it in an alcohol/water solution and the resulting slurry was spread on absorptive paper. This sample is representative of the distribution of fragment sizes making up the comb.

Leaf discs and fragments from the comb were taped onto watercolour paper, labelled and photographed or scanned onto a computer (figure 2*a*,*b*). The leaf fragments were separated from each other by placing pieces of comb into ethanol solution (one part ethanol two parts water) before spreading the fragment slurry onto absorptive watercolour paper. The fungal substrate from Minca remained in ethanol for several months before being spread onto paper, while the Tiputini substrate was only in the solution for a few hours. We have previously measured shrinkage of *Odontadenia macrantha* leaf discs taped to watercolour paper, finding a perimeter decrease of 2.5% over the course of a year [14]. We tested for size changes caused by the ethanol treatment and for shrinkage of taped leaf fragments. The results are included in the error analysis below.

The surface area and perimeter were measured for every disc and fragment, using Image J, version 12.0.0 analysis software [33], and averages were calculated for each colony. Original leaf edges were identified under a microscope by looking for a continuous surface layer, and marked to avoid inclusion of natural edges in cutting estimations. The average number of fragments cut from a foraged leaf disc was estimated as the ratio of the average surface area of the leaf discs over the average surface area for the comb fragments. This value was multiplied by the average leaf fragment perimeter, and the average total leaf disc perimeter was subtracted so it would not be counted as cutting in the nest. The value was divided by two, because each cut during disc reduction leads to two new fragment edges. The average cutting distance for one disc (cut inside the nest) was divided by the average leaf disc perimeter (cut outside the nest) minus natural edges, giving us a ratio that estimates the amount of cutting above-ground versus underground. The result is the following equation:

2.1 $$\frac{\text{cut distance inside the nest}}{\text{cut distance outside the nest}}≅\frac{0.5((A_{\mathrm{disc}}\;P_{\mathrm{fragment}.\mathrm{total}}\text{/}A_{\mathrm{fragment}})-P_{\mathrm{disc}.\mathrm{total}})}{P_{\mathrm{disc}.\mathrm{cut}}},$$

where *A* and *P* stand for the average surface area and perimeter, respectively, of fragments or discs as indicated by the subscript, which also indicates whether the perimeter value includes the total perimeter or the part of the perimeter that was actually cut (not including natural edges).

To estimate the cutting density, the product of the average leaf fragment perimeter and the number of fragments prepared from a leaf disc was added to the average leaf disc perimeter (minus natural edges) to give us the total cutting distance per leaf disc. This number was divided by the average leaf disc surface area to give us an estimate of the metres of leaf cut per square metre of leaf.

2.2 $$\text{cutting density}≅\frac{0.5((A_{\mathrm{disc}}\;P_{\mathrm{fragment}.\mathrm{total}})\text{/}A_{\mathrm{fragment}})-P_{\mathrm{disc}.\mathrm{total}})+P_{\mathrm{disc}.\mathrm{cut}}}{A_{\mathrm{disc}}}.$$

### 2.3 Error analysis

The errors reported in table 2 include, where appropriate, contributions from the uncertainty of the mean, errors associated with selecting a minimum fragment size threshold, image digitization and measurement, variations in the shapes of the discs and fragments, and size changes due to preservation in ethanol or from desiccation. The largest source of error was due to the standard deviation of the means because of the large variation in sizes of leaf discs and fragments in the comb.

The error for image digitization and measurement was estimated to be 0.9% for perimeter and 2% for area by comparing results for three different photos of the same set of leaf discs and fragments.

The lower fragment size threshold (0.0005 cm^2^) was set from video observations of the smallest fragments incorporated into the comb (table 1). This threshold was set to avoid small pieces of leaf material resulting from decay of fragments in the comb. To estimate the uncertainty associated with the use of this threshold, we recalculated cutting density using a threshold of 0.00005 cm^2^, which resulted in a 1.4% or 2.8% increase for perimeter and area, respectively.

**Table 1.** Observations of leaf processing in an *A. cephalotes* laboratory colony following the delivery of a new leaf disc to the fungus garden. The behaviours are ordered according to their typical first appearance during processing. Only studies that examined *Atta* spp. are included in the reference column of this table.

behaviour (synonyms used in the literature)	description	short defining video clip	long video clip	references
compilation of behaviours		all short clips^a^		
holding (hoisting)	mandibles are used to lift and stabilize a leaf disc or a fragment that other ants are processing	holding short^b^	holding long^k^	[17,22,26,29,30]
licking (rasping)	the surfaces and edges of a leaf disc or fragment are contacted with the glossa and palps	licking short^c^	licking long^l^	[15–18,20–22,29,30]
scraping	less common: the mandibles are actuated repeatedly at or near the leaf surface during licking	scraping short^d^	scraping long^m^	[30]
cutting (shredding)	mandibles are cut either by dragging one mandible through the leaf, or by a symmetrical two-mandible technique	cutting short^e^	cutting long^n^	[15–18,20–22,26,29,30,32]
puncturing (chewing, crimping, masticating, scarring, pressing)	the edge of a leaf fragment is held between the mandibles, which are closed to puncture both sides with the mandibular teeth	puncturing short^f^	puncturing long^o^	[16–18,20–22,29,30]
adding abdominal emissions (depositing faecal fluid)	less common: abdominal excretion is deposited or transferred onto a fragment being processed	adding abdominal emissions short^g^	adding abdominal emissions long^p^	[22,29,30]
caching fragments	a fragment is temporarily set down in a region of the comb with other fragments	caching short^h^	caching long^q^	
inserting fragments	a leaf fragment is inserted in the fungal comb while the fragment is rocked from side to side	inserting short^i^	inserting long^r^	[15,16,20–22,29,30,32]
inoculating fragments	fresh hyphal tufts are plucked from older comb and attached to or between leaf fragments that have recently been incorporated	inoculating short^j^	inoculating long^s^	[15,17,20,21,29,30]

^a^https://www.youtube.com/watch?v=A9sYr1fsBAE

^b^https://www.youtube.com/watch?v=dzUGhJ3Alnw

^c^https://www.youtube.com/watch?v=WHp8hyiWdtY

^d^https://www.youtube.com/watch?v=eN39nBsDcfU

^e^https://www.youtube.com/watch?v=dBp_K1V7xVQ

^f^https://www.youtube.com/watch?v=7IpxNctpSt0

^g^https://www.youtube.com/watch?v=lxQ-4fCzStc

^h^https://www.youtube.com/watch?v=lKIOIVKlmrc

^i^https://www.youtube.com/watch?v=NSx3_JJtjLo

^j^https://www.youtube.com/watch?v=0wdC4PhcEY8

^k^https://www.youtube.com/watch?v=CjR73m3CkHc

^l^https://www.youtube.com/watch?v=pZu00swB47A

^m^https://www.youtube.com/watch?v=Y1EqY9EAFts

^n^https://www.youtube.com/watch?v=kWO80LZq44k

^o^https://www.youtube.com/watch?v=0MqPLExoBnw

^p^https://www.youtube.com/watch?v=Uff7ngmWEt4

^q^https://www.youtube.com/watch?v=XW3kAphvZMY

^r^https://www.youtube.com/watch?v=j2g15h_urZQ

^s^https://www.youtube.com/watch?v=A39B2m25edE

The effect of the ethanol/water mixture on leaf fragments from Minca was estimated by observing changes in fresh fragments from our laboratory colony after a month of soaking in the solution (area, 4%; perimeter, 2%; three batches of 15 fragments, area s.d., 3%). Shrinkage of collected leaf discs taped to paper was measured to be 4.8% in area after drying.

The methods we used for estimating cutting ratio and cutting density (equations (2.1) and (2.2)) are accurate and not just estimates when applied to individual discs and the specific fragments from those discs. However, since the fragments in the comb are no longer associated with the originating discs, we use average values of area, perimeter, and natural edges in equations (2.1) and (2.2), making an estimate by assuming that all discs and all fragments are the same size and shape. To investigate the error introduced by this assumption, we calculated the standard deviation of the ratio of perimeter squared to area (an indicator of aspect ratio) for the discs and for the fragments. We then recalculated cutting ratio and density using values obtained by increasing or decreasing the means used in the calculations by 1 s.d. of the mean. That is, we re-calculated assuming disc or fragment length-to-width ratios that were further from or closer to a circular shape by 1 s.d. The resulting error estimate was 9% for Minca and 4% for Tiputini.

### 2.4 Testing preference for pre-cut leaves

**Figure 3.**
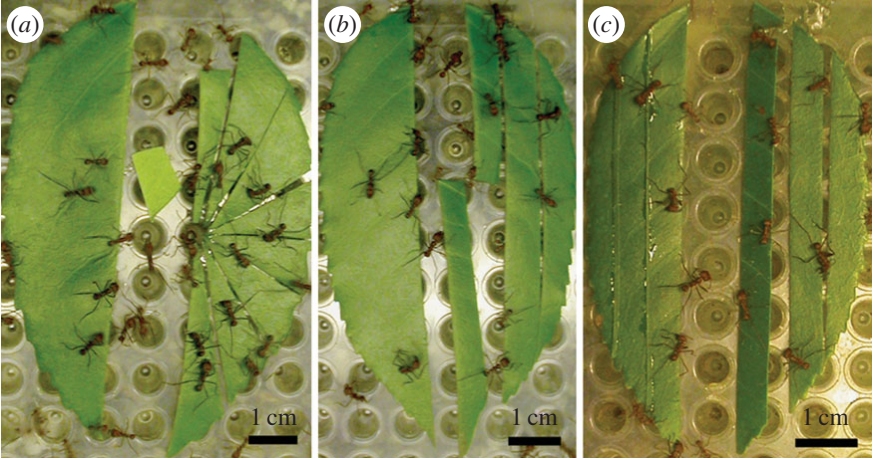
More ants accumulate on leaf halves that have been subdivided. (*a*,*b*) Radially and linearly subdivided halves, respectively. (*c*) Linearly divided half with a full half that has added cut strips to equalize wound area. Photographs have been selected that have, approximately, the average number of ants for each treatment in table 3 (Leaf Preference Results).

Mature, healthy and symmetric *P. lusitanica* leaves were cut to excise the central vein. The two resulting pieces were trimmed to equal weight (approx. equal area), and one side (alternating between left and right) was cut into either eight radial sections or, to test another fragment geometry, into 0.5 cm wide longitudinal strips (see figure 3*a*,*b*, respectively). The whole and cut halves were attached to 96-well PCR plates with a single drop of epoxy at the stem side of uncut halves and longitudinally cut pieces, or at the thinnest point of radially cut pieces.

The plates with leaf pieces were placed on the laboratory foraging platform. Immediately after an ant cut out the first complete disc from any of the pieces on the plate, three photos were taken about 5 s apart. In each of the three photos, ants were counted as being on a piece if the maxillae were either located directly above or else touching leaf material, such as in cutting or grasping. The average numbers of ants present on the full half were compared to the numbers on the subdivided halves to see whether there was a bias towards the smaller pieces. To control for the possibility that ants were recruited to greater wound area and not to smaller pieces, we altered the above procedure for longitudinally cut pieces by adhering 1 mm wide strips to the full halves using drops of epoxy (Quik-Cure 5 min) at 5 mm intervals (figure 3*c*). In this way, the wound area per unit leaf area was about the same on the full half and the cut-up half. To control for the possibility that the epoxy that was used to adhere the wounded tissue to the leaf half affected recruitment, we also compared halves with epoxy, but no wounded tissue, to untreated halves.

## 3. Processing observations and discussion

Table 1 contains a summary of our processing observations and links to short (less than 10 s) or longer videos demonstrating each of the behaviours.

### 3.1 Holding

One or more ants in the fungus garden held a leaf disc or fragment in the air, stabilizing it with mandibles and sometimes with legs, while other ants engaged in the processing behaviours below. Ants that hold leaf discs or fragments might reduce movement and provide tension for cutting or other processing behaviours. This cooperation may reduce energy expenditure.

The ‘Holding’ videos illustrate holding when it was the sole activity of an ant. A second type of holding was typical when the fragments were smaller and a single ant both held and processed the fragment. Illustrations of this type of holding are found in the videos of the primary task: licking, cutting, puncturing, etc. The lone ant would hold, rotate and manipulate the fragment in a manner that has not previously been described, with the tarsi and tarsal claws of three of its legs, the two forelegs and either the left or right middle leg. The remaining three legs formed the tripod that supported the ant.

The tarsi appear to be prehensile and segments of a tarsus would often curve around the edge of a leaf fragment, maintaining close contact (see, for example, the last clip in the ‘Licking long’ video). The tarsal claws of the three holding legs often played a role as well, catching the fragment edges or surface like grappling hooks.

### 3.2 Licking

Ants contacted the leaf disc or fragment with glossa and palps, apparently covering the entire surface. The ants gave particular attention to freshly cut leaf edges, which tended to blacken later (leaf edges did not blacken when cut with a scalpel). This behaviour takes place on the uncut surface of leaves and therefore does not involve the ingestion of sap, although the term ‘licking’ has been used elsewhere to describe potential sap ingestion [18]. The antibiotic and antifungal properties of metapleural and mandibular gland secretions have been demonstrated in previous investigations of leafcutters [34–39]. It has been suggested that these secretions are transferred to the substrate during licking, where they promote aseptic conditions that benefit *L. gonglyophorus* [40,41]. It has also been suggested that licking is involved in the removal of epicuticular waxes [19,27,30].

### 3.3 Scraping

While the glossa and palps were in contact with the leaf surface (during licking) the mandibles were sometimes opened and closed repeatedly with the tips of the distal teeth appearing to barely contact the surface of the leaf. We have not examined the scraped surface with enough resolution (e.g. SEM) to determine whether a surface layer of the leaf is sliced. If so, scraping may increase fungal access [42]. Alternatively, scraping may remove foreign material from the leaf surface. This behaviour has also been implicated in the removal of epicuticular wax [30].

### 3.4 Cutting

Cutting with the mandibles (which start off as sharp as razor blades [14]) was the only observed means by which the leaf discs were reduced to the size of fragments in the comb (0.0005–0.04 cm^2^). This in-nest cutting was accomplished using the same two techniques that have been observed outside the nest [14]. In the anchor and drag technique, the leading mandible pierces the leaf and provides a nearly fixed anchor as the ant drags the lagging mandible through the leaf by closing its mandibles. At the same time, the ant may also pull or push the cutting mandible with its head and body. When cutting was progressing slowly, the leading mandible was used to slit a surface layer of the leaf along the cutting path and then used as an anchor for the lagging mandible, which was then dragged through the partially cut leaf. For the second technique, used for larger veins and shorter cutting distances, mandibles were actuated symmetrically in a slicing or sawing motion.

### 3.5 Puncturing

During the puncturing process, the edge of the fragment was held between the mandibles as they were repeatedly closed, piercing both sides of the fragment with the mandibular teeth. Following puncturing, many puncture marks and surrounding regions darkened visibly over a period of about 10 min (puncture darkening (https://www.youtube.com/watch?v=sPl25NqTG4E)). It has been suggested that the darkening results from oral secretions [17]. We did observe glossal contact during puncturing, but only occasionally (see ‘puncturing long’). A second possibility is that the intramandibular glands that secrete onto the surface of the mandibles [43] are responsible for the darkening. Puncturing may facilitate entry of both the darkening secretion and the fungus into the leaf interior. It may be that the maximum leaf fragment size in the comb is such that the entire surface of the fragment can be punctured because no region is more than a mandible length away from the edge.

### 3.6 Adding abdominal emissions

Abdominal emissions (often referred to with the more specific term ‘faecal fluid’) have long been associated with *Atta* nest founding and substrate preparation activity [6,29,44]. Deposition of abdominal emissions onto leaf fragments during processing has been reported as common in certain accounts of *Atta* processing behaviour [30] and uncommon in other accounts [22,28,29]. We observed the deposition of abdominal emissions infrequently relative to other processing behaviours and we are not certain that every fragment received this treatment before incorporation into the comb. Most observed instances occurred while the ant was puncturing the fragment (see abdominal emission videos linked in table 1). Ants displayed a remarkable behavioural plasticity in accomplishing this task: in two of the three videos, the abdominal excretion is deposited directly on the fragment. In one video, the droplet is transferred from the abdomen to a foreleg, then to a mandible and finally to the leaf fragment.

Many studies have investigated the composition of fluid extracted from leafcutter ant abdomens [39,45–56] and discussed potential benefits (e.g. leaf decomposition) of applying components of the fluid to the fungal garden or to fragments during leaf processing. However, as a cautionary note, these studies obtained fluid by artificial methods and we are not aware of studies that sampled fluid that had been deposited during leaf processing. In our observations, we could not be sure that the fluid applied to the fragments was faecal fluid and not glandular secretions or a mixture of both.

### 3.7 Caching fragments

We observed fragment caching within the nest, which was similar to leaf disc caching behaviour witnessed outside of the nest during foraging [3,57,58]. To the best of our knowledge, this is a previously unreported example of task partitioning between individuals in *Atta* [59].

### 3.8 Inserting fragments

The fragments were inserted into the comb structure by rocking them into place, much as a stonemason rocks stones into place in a rock wall [16]. Rocking may not only leverage the fragment into the comb wall but may also provide mechanical feedback: rocking probably requires more force as the fragment is securely incorporated into the comb.

On a couple of occasions, we have observed ants pull and reinsert fragments that had been recently inserted. At least in one case, the fragment that was reinserted appeared to have been loose (see last clips in ‘Inserting long’ video and associated description), suggesting the possibility of a ‘quality control’ or ‘repair’ task.

### 3.9 Fragments, not pulp

The fully processed leaf material that was inserted into the comb differed from that described in much of the literature [2,3,15,16,19,24,52,54,56,60–69]. While observing our laboratory colony and examining wild colony garden material, we have exclusively encountered leaf fragments that were relatively intact: wider than the thickness of the leaves and not folded or crushed (see videos linked in table 1). We did not observe insertion of pulp into the comb, and the filtered slurries of fresh (generally from the top) comb material from wild colonies did not contain pulp, only leaf fragments. Our observations are, therefore, not consistent with descriptions of pulp, crushed pellets or balls of leaf that many authors have mentioned for observation of *Atta* and *Acromyrmex*. Reports of crushed pellets or balls may be associated with extremely thin leaves or petals, or misinterpretations of the balls of fungus that are plucked in fungal-rich areas and packed into the comb between new leaf fragments. The distinction between pulp and the relatively intact fragments that we have observed is important because reducing the fragments into a pulp would greatly increase the energy cost of leaf processing. The final size of the fragment may be an optimal balance: smaller fragments probably increase fungal and enzymatic accessibility but cost more energy.

### 3.10 Inoculating fragments

Small ants planted clumps of fungus that had been plucked from fungus-rich areas. The fungus was planted either into leaf fragments that had been incorporated into the comb, or between the fragments in the comb. The video clips linked in table 1 show that the fungus is planted along the exposed edge of a fragment (possibly secured in puncture notches) as well as into punctures that the ants had made in the centre of the fragment and, second, in larger balls of fungus stuffed between fragments. The ants pushed the fungus into place with the tarsi of their front legs. For large clumps inserted between leaf fragments, they rocked the ball of fungus into place like they did with leaf fragments. These large balls of fungus may serve as mortar, filling spaces between fragments and stiffening the wall, as well as inoculating it.

In summary, the complexity of the processing behaviours that emerge from these miniature brains, and the additional complexity associated with their behavioural plasticity, seems, to us, to support the potential use of ants as models for neurological studies of behaviour [70], and also suggests that leafcutter ants may be valuable models for studies of behaviourally related gene expression [71,72].

## 4. Cutting density and ratio results

Table 2 shows that, for a square metre of leaf material collected by *A. cephalotes* or *A. colombica*, about 2900 m of cutting takes place, with about 350 m of cutting outside the nest and about 2550 m of cutting within the nest.

**Table 2.** Area and perimeter averages and resulting estimates of inside/outside cutting ratios and cutting density (errors are the calculated 67% CIs and not the standard deviation of the sample values).

	*Atta colombica*, Tiputini (mature colony)		*Atta cephalotes*, Minca (2-year-old colony)	
	foraged leaf discs	leaf fragments from comb	foraged leaf discs	leaf fragments from comb
surface area	0.99±0.056 cm^2^ (*n*=144)	0.0055±0.00038 cm^2^ (*n*=247)	0.52±0.036 cm^2^ (*n*=94)	0.0044±0.00038 cm^2^ (*n*=164)
cut perimeter {total–natural edges}	3.21±0.13 cm {3.83–0.62 cm} (*n*=144)	0.29 cm ±0.010 cm (*n*=247)	1.87±0.11 cm {2.96–1.09 cm} (*n*=94)	0.25±0.011 cm (*n*=164)
ratio of cut distance, inside/outside-nest	7.50 ±0.57		6.90 ±0.78	
cutting density	2869±222 m m^−2^		2994±352 m m^−2^	

To what degree can these results for two colonies at specific times be generalized to other *Atta* colonies? If we assume that the cutting densities and ratios are normally distributed among colonies and over time, and use no prior information, Students *t*-statistic for a sample size of two suggests that, at a 95% confidence level, the cutting density mean for *Atta* is between 2.1 and 3.7 km m^−2^, and the inside/outside ratio of cutting is between 3.4 and 11.2. These uncertainty bands would be narrowed if we incorporated prior information that restricted, for example, the maximum possible size of fragments in the comb.

The data here also allow us to test a simplified estimate of cutting density that can be applied to any colony with a known average area of fragments in the comb. Assuming that the final fragments are identical squares and that the cutting distance is much greater than the perimeter of the leaf or plant structure, the cutting density in metres cut per square metre of leaf would be estimated from the average area of a comb fragment as:

4.1 $$\text{cutting density}≅2+\frac{2}{\sqrt{A_{\mathrm{fragment}}}}.$$

This estimate predicts a cutting density of 2699 m m^−2^ for the first colony, 0.94 of the value for the more detailed estimate in table 2, and 3017 m m^−2^, for the second colony, 1.008 times greater than the value in the table.

The success of this simple estimate demonstrates that the plant material reduction geometry is simple, and thus likely to be similar in other colonies.

For the cutting ratio, we use the assumptions above and further assume that the discs transported to the nest are uniform squares. The ratio of cutting inside over cutting outside would be

4.2 $$\frac{\text{cut distance inside the nest}}{\text{cut distance outside the nest}}≅0.5\left(\sqrt{\frac{A_{\mathrm{disc}}}{A_{\mathrm{fragment}}}}-1\right).$$

The values from this estimate were 0.83 and 0.72 of the values in table 2, probably not as accurate as the cutting density estimate because a significant fraction of the disc perimeters were not cut but were natural edges (table 2).

## 5. Pre-cut leaf preference results

We conducted 20 presentations for each of the experiments and compared the number of ants on equivalent areas of cut up and control leaf pieces as an indicator of preference. More ants were present on cut up leaf halves than on whole leaf halves (table 3 and figure 3). The ants preferred the smaller pieces even when the wound area was equalized. The subdivided leaf halves were cut into discs significantly faster than the whole halves (60 out of 60, *p*<1×10^−18^), since more ants were involved in cutting and less cutting was needed than for the whole pieces.

**Table 3.** Ants prefer subdivided leaf halves over full halves.

test	recruitment to full leaf halves (no. ants±s.d.)	recruitment to leaf halves that have been subdivided (no. ants±s.d.)	*n*^a^	*p*-value (Wilcoxon two (sample test)
linear sub-division test	7±3	12±4	20	0.0003
radial sub-division test	9±3	14±5	20	0.0001
equal wound, linear sub-division test	6±2	10±2	20	0.0007
epoxy control for extra epoxy on the full half of the equal wound test	$\frac{\mathrm{noextra}}{6\pm 4}\;\frac{\mathrm{extra}}{8\pm 3}$		20	0.26

^a^number of presentation experiments.

## 6. Discussion

We have previously estimated that the energy expended cutting *Odontadenia macrantha* leaves into discs for transport to the nest was about 1.3×10^4^ J kg^−1^ for ants with pristine mandibles from a colony that we studied in Panama [14]. A rough estimate of the total cutting cost can be made by assuming that the energy cost of cutting per metre of cut is the same in the nest as it was outside. Thus, we multiply the estimated cost for external cutting by 7, resulting in a total estimated cost for cutting outside and inside the nest of about 1×10^5^ J kg^−1^. Typical combustion energy densities in leaves are in the range 0.3−3.0×10^6^ J kg^−1^ (varying mainly with water content) [73,74]. Considering that metabolic efficiency is about 40% and the efficiency of converting mulch calories into commercial fungus calories is about 60% [75], the calories available from the fungus are likely to be around 2.4×10^5^ J kg^−1^ of original 1×10^6^ J kg^−1^ leaf material. We estimate the energy cost of transport from Lighton *et al.* [12] along with Schofield *et al.* [14] to be about 1.5×10^3^ J m^−1^ kg^−1^. At 100 m from the nest, the carrying cost would be 1.5×10^5^ J kg^−1^, and the combined cutting and carrying cost would be about 2.5×10^5^ J kg^−1^, just exceeding the estimate of the energy available from the fungus grown on 1×10^6^ J kg^−1^ leaf material. This rough estimate suggests that leafcutter ants may be operating with slim energy profit margins, and that energy conservation may be an important selective pressure. Better estimates of the in-nest processing cost and better measures of the efficiency of converting leaf calories into fungal calories will lead to improved estimates of the energy profit margin.

The estimate that cutting costs are comparable to transport costs, and the observation that most cutting takes place within the nest, suggest that in-nest processing costs must be considered in foraging energetics [26,76]. In the light of the fact that plant material energy densities vary by at least an order of magnitude, the energy content of plant materials should be considered in studies of ant preference, or, more precisely, the ratio of combustion energy density to cutting energy density (the energy required to cut a kilogram of the material into comb fragments).

For two equivalent materials, the cutting energy density is lower if the ratio of natural perimeter to mass is greater: smaller leaves would require less cutting because there is more natural edge. Our pre-cut leaf test indicated that more ants per unit area are recruited to smaller leaf pieces. However, the cutting energy density advantage would be small unless the leaves or leaf portions were very small, having an area within an order of magnitude or so of the comb fragment area. It may be that, instead of cutting energy density, there is an advantage in selecting smaller pieces because the time spent in the dangerous environment outside of the nest is reduced. This time advantage becomes important when the leaf area is within an order of magnitude of the transported disc area, not the comb fragment area.

It has been reported that the youngest ants, callows, do not typically appear outside the nest [20]. This research supports the possibility that the nest phase extends well into adult life because most cutting takes place within the nest. In a recent study, Schofield *et al.* [14] found that the mandibles of *A. cephalotes* collected outside of the nest were highly worn. Ants cutting outside the nest were estimated to take, on average, twice as much time and energy to cut leaves as ants with pristine mandibles. The ants with the most worn mandibles did not even cut, but carried instead. The extra energy cost of cutting with worn mandibles suggests that it may be most efficient for ants with sharp mandibles to stay safely in the nest. Leafcutter ants may spend the first portion of their lives underground, while their mandibles are sharpest, both avoiding risk and efficiently processing the endless stream of leaf discs.

## Data accessibility

We have made all of our data publicly accessible. The data are spread over three archives available at the following locations: https://dx.doi.org/10.6084/m9.figshare.2055933.v1, https://dx.doi.org/10.6084/m9.figshare.2055945.v1 and https://dx.doi.org/10.6084/m9.figshare.2055948.v1. All spreadsheets, images and videos have been included. We amassed approximately 90 GB of processing behaviour footage during the course of this study. Contact the corresponding author if you are interested in viewing or obtaining additional video that was not incorporated into the final manuscript.
